# ER-CGKA: Efficient and robust continuous group key agreement scheme with post-compromise forward security for IoV

**DOI:** 10.1371/journal.pone.0307867

**Published:** 2024-08-29

**Authors:** Guishuang Xu, Xinchun Yin, Xincheng Li

**Affiliations:** 1 College of Information Engineering, Yangzhou University, Yangzhou, Jiangsu, China; 2 Henan Key Laboratory of Network Cryptography Technology, Infomation Engineering University, Zhenzhou, Henan, China; 3 Guangling College, Yangzhou University, Yangzhou, Jiangsu, China; University of Lagos Faculty of Engineering, NIGERIA

## Abstract

The Internet of Vehicles (IoV) counts for much in advancing intelligent transportation by connecting people, vehicles, infrastructures, and cloud servers (CS). However, the open-access wireless channels within the IoV are susceptible to malicious attacks. Therefore, an authentication key agreement protocol becomes essential to ensure secure vehicular communications and protect vehicle privacy. Nevertheless, although the vehicles in the group are compromised, they can still update the group key and obtain the communication content in the existing group key agreement protocols. Therefore, it is still challenging to guarantee post-compromise forward security (PCFS). Dynamic key rotation is a common approach to realizing PCFS, which brings a heavy computation and communication burden. To address these issues, an efficient and robust continuous group key agreement (ER-CGKA) scheme with PCFS is designed for IoV. The propose-and-commit flow is employed to support asynchronous group key updates. Besides, the computation cost and communication overhead are significantly reduced based on the TreeKEM architecture. Furthermore, we adopt the threshold mechanism to resist the collusion attacks of malicious vehicles, which enhances the ER-CGKA scheme’s robustness. Security analysis indicates that the proposed scheme satisfies all the fundamental security requirements of the IoV and achieves PCFS. The performance evaluation results show that our ER-CGKA scheme demonstrates a reduction in the computation cost of 18.82% (Client) and 33.18% (CS) approximately, and an increase in communication overhead of around 55.57% since pseudonyms are utilized to achieve conditional privacy-preserving. Therefore, our ER-CGKA scheme is secure and practical.

## 1 Introduction

### 1.1 Motivation

The Internet of Vehicles (IoV) integrates advanced sensors and modern communication technology to optimize driving efficiency and solve traffic problems (e.g., traffic jams and frequent traffic accidents). In IoV, each vehicle is equipped with a wireless communication device called an on-board unit (OBU), which is used to communicate with other vehicles and roadside units (RSU). There are two communication modes in IoV, i.e., vehicle-to-vehicle (V2V) and vehicle-to-infrastructure (V2I) communications [1]. According to cellular vehicle-to-everything (C-V2X) [2], vehicles broadcast real-time traffic information (e.g., location, speed, weather information, etc.) every 100 milliseconds. However, security threats and privacy disclosure are two obstacles hindering the application of IoV [3]. On the one hand, IoV is quite vulnerable to attacks due to open-access wireless channels. As the vehicles usually drive at high speed, if the attacker injects an attack instructions at the device terminal or cloud server (CS), it is likely to endanger the lives of drivers and passengers. On the other hand, the attacker can eavesdrop and sniff the transmitted traffic messages, which leads to serious disclosure of users’ privacy.

Group key agreement (GKA) is one of the effective ways to solve the above problems, where vehicles beyond the group cannot send encrypted messages to group members [4]. Besides, it allows multiple group members to fairly negotiate a secret group key in open networks, which enables subsequent secure communications. Nevertheless, there are some shortcomings in existing GKA protocols. First, they cannot guarantee post-compromise security (PCS) due to the lack of an effective revocation mechanism, and the corrupted vehicles can update the group key all the time. Second, vehicles in the same group may collude with each other for more benefits. They would send malicious or fake messages to the group and disclose the group key to attackers, which seriously disturbs the communication security of the group [5]. Finally, vehicles and servers must remain online and conduct several interactions to rotate the group key. Since the rotation may be frequent, the process is extremely inefficient.

In recent years, continuous group key agreement (CGKA) [6] has provided a powerful abstraction to reason on the above security requirements. CGKA implements group operations using O(log(*n*)) computation, communication, and storage with the TreeKEM protocol [7]. The propose-and-commit flow is employed to provide group key rotation for the next epoch. Therefore, the CGKA protocol achieves post-compromise forward security (PCFS) efficiently. It also achieves asynchronous decentralized group key management for large dynamic groups using a CS without disclosing group key information. However, the fundamental CGKA protocol cannot satisfy security and privacy-preserving properties in specific applications (e.g., traceability and privacy-preserving in IoV). Therefore, an efficient and robust continuous group key agreement (ER-CGKA) scheme extending Hashimoto et al.’s work [8] is proposed for IoV to provide asynchronous GKA and PCFS. The proposed ER-CGKA can not only inherit the advantages of CGKA but also satisfy the security and privacy-preserving requirements of the IoV.

### 1.2 Contribution

The main contributions of the work are shown as follows.

1. **An efficient and robust continuous group key agreement (ER-CGKA) scheme is proposed to resist collusion attacks.** The protocol extends a metadata-hidden CGKA scheme and provides robustness against collusion attacks among group users. Threshold cryptography is leveraged to guarantee that adversaries less than the threshold cannot disturb the group key management and threaten group communications.
2. **It is the first time that the CGKA protocol is applied to IoV to provide PCFS.** In ER-CGKA, vehicles are distributed in a pool of pseudonyms to achieve privacy-preserving and traceability at the same time. Traffic management center (TMA) can recover the real identity of malicious vehicles through pseudonyms, while other entities cannot identify vehicles.
3. **The proposed ER-CGKA scheme is secure and efficient.** Security analysis indicates that it satisfies all the fundamental security requirements like conditional privacy-preserving, confidentiality, unlinkability, PCFS, and resistance to collusion attacks. The performance analysis shows that ER-CGKA has low computation costs and communication overhead, which is suitable for resource-constrained IoV scenarios.

**Fig 1:**
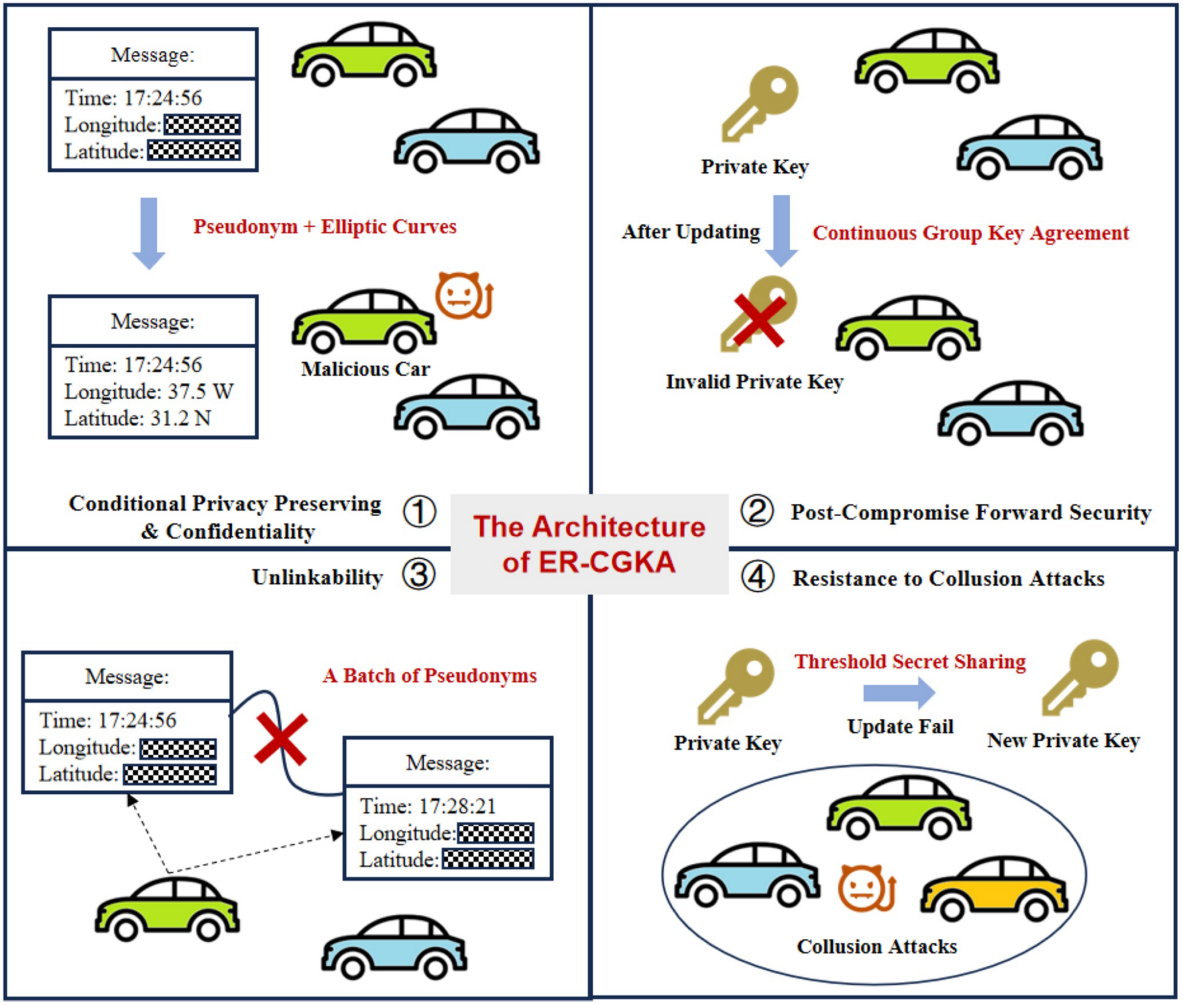
The architecture of the work.

The architecture of the work is shown as Fig 1. The functions of the work mainly include four parts. Prat one is conditional privacy-preserving and confidentiality, which is achieved by pseudonym technology and elliptic curve cryptography (ECC) assumption. Part two is the PCFS guaranteed by the treeKEM protocol. Part three is unlinkability, which is ensured by a batch of pseudonyms. Part four is the resistance to collusion attacks, based on a (*k*, *n*) secret-sharing scheme.

### 1.3 Organization

The rest of the paper is organized as follows. Section 2 surveys the related work. In Section 3, preliminaries are introduced. In Section 4, the ER-CGKA scheme is presented in detail. Section 5 demonstrates the security proof and analysis under the random oracle model (ROM). The performance evaluation is given in Section 6. At last, we conclude the work in Section 7.

## 2 Related work

### 2.1 Synchronous group key agreement

Many synchronous GKA protocols for IoV have been proposed to construct secure vehicular communications. Since all vehicles in the group should contribute to the new group key, several rounds of interactions between them are necessary. Though Boneh and Silverberg [9] leveraged the military mapping technique to reduce it to one round, the computation cost is high, and an additional round of communication is required for key authentication. Meanwhile, Hu et al. [10] pointed out that military mapping is insecure, which requires all group members to be online at the same time. However, it is unrealistic due to the time difference that exists among group members.

The authenticated key agreement (AKA) schemes are proposed to ensure message transmission security and the privacy of vehicles. Canetti and Krawczyk [11] pointed out that a secure key agreement protocol can be designed with a session key security algorithm, symmetric encryption algorithm, and authentication algorithm. Dua et al. [12] devised a lightweight AKA scheme based on ECC. However, a trusted cluster vehicle is required in their scheme, and the group key updates are not considered. Islam et al. [13] designed an AKA scheme based on a finite field without ECC or bilinear pairing (BP) operation. However, their scheme has some shortcomings. First, TMA needs to be online during group key updates and unicast the new group key to each vehicle, which is inefficient and has poor application. Then, it simply performs the “XOR” operation on the updated key and the old key to complete the encryption operation of the new group key, and the encrypted updated key is exposed in the public channel. Therefore, the vehicle that has been removed from the group can still compute the updated key. Ma et al. [14] devised an AKA scheme based on fog computing and proved its security under the ROM, but the costs of computation and communication are still high. Cui et al. [15] proposed an AKA scheme under the multi-cloud environment based on ECC. Although the computational efficiency has improved since not using BP, single-TMA makes the scheme vulnerable to denial of service (DoS) attacks. Wei et al. [16] raised a lightweight AKA scheme supporting conditional privacy-preserving based on the multi-TMA model, which employed the technologies of Lagrange interpolation theorem, symmetric encryption, hash function, and pseudorandom function (PRF). Their scheme can resist DoS attacks to some extent but relies on trusted TMA to perform group authentication.

In 2014, Karuppiah et al. [17] proposed a secure remote user mutual authentication scheme using smart cards, which enables the user to choose his/her password freely and renew the password anytime. Based on Chinese remainder theorem, Vijayakumar et al. [18] designed a centralized dynamic group key management for secure multicast communication. The the computation complexity of the key server is reduced to O(1). In 2015, Vijayakumar et al. [19] proposed a dual AKA technique for secure data transmission in vehicular ad hoc networks (VANET), adding or revoking users in the VANET group can be performed in a computationally efficient manner by updating a small amount of information. For secure authentication in global mobility networks, Karuppiah et al. [20] designed an anonymous authentication scheme for roaming service, providing perfect forward secrecy and detect wrong passwords quickly. In 2016, Karuppiah et al. [21] put forward a lightweight authentication scheme with user anonymity, providing local password verification, and resisting replay attacks. In 2017, Karuppiah et al. [22] proposed a dynamic ID-based generic framework for an anonymous authentication scheme, which offers the user anonymity, password change, updating option, and quick detection of wrong password. In 2023, Tan et al. [23] designed an AKA scheme for UAV-assisted infrastructure-less IoV. They deployed the tethered UAV (TUAV) as the specific mobilized base station so that the active edge IoV infrastructure is not needed. Based on chaotic maps, Zhang et al. [24] proposed a sustainable AKA protocol for industry 5.0, which can achieve secure authentication among consumers, gateway nodes, and Internet of Things-based consumer electronics and generate session keys. To decrease the frequent interaction of vehicles with the TMA, Saleem et al. [25] proposed a secure access control protocol with conditional privacy for VANET, which does not require the TMA’s involvement during authentication between vehicles and RSUs. Soon after, a privacy-preserving AKA protocol for VANET using the hashing technique was proposed [26], which provides an efficient and secure data transmission mechanism over a public communication channel. Mahmood et al. [27] developed a key agreement solution for mobile users to realize mutual authentication in a single round. Their protocol offers user anonymity and prevents physical attacks by physically unclonable functions. This year, Manickam et al. [28] designed a three-factor mutual authentication scheme for telecare medical information system based on ECC, which protects sensitive patient data from getting out during communication and protects against different types of security attacks. Saleem et al. [29] designed a hash-based authentication scheme, which effectively combines identity, password, and bio-metric to enhance resistance against impersonation, denial of service, and privileged insider attacks. Gautam et al. [30] suggested a blockchain-based authentication framework for intra-twin and inter-twin communication in vehicular digital twin networks and the integrated blockchain in the system assures data compactness and verifiability.

Since all vehicles in the group should contribute to the new group key, several rounds of interactions between group members are necessary. Therefore, the synchronous group key agreement schemes have rigorous requirements for latency and robustness of IoV.

### 2.2 Asynchronous continuous group key agreement

To improve communication efficiency and achieve PCFS, the CGKA protocols are proposed. In 2019, Weidner et al. [31] raised that updating randomized key information instead of overwriting, cannot achieve forward security (FS) and lacks formal security proof. In 2020, Alwen et al. [6] analyzed the TreeKEM [7] scheme and pointed out that it cannot satisfy PCFS security requirements. Then, they first proposed the concept of CGKA, which achieves key updates that are invisible to CS in different states with a renewable encryption technique. However, neither of their models supports concurrency, nor prevents message injection by malicious attackers. Later, a stronger adversary model was put forward and the concept of real-or-random was first introduced [32]. The definition in [32] gives the attacker the ability to be highly adaptable and fully proactive, corrupting any participant, and even arbitrarily setting the random numbers required for key agreement. Simultaneously, the robustness of CGKA is improved with zero-knowledge proofs (ZKP). In 2021, Klein et al. [33] first proposed a CGKA protocol that can guard against adaptive adversaries. Soon after, the history graph [34] was invoked to describe the syntax of CGKA and first provides a black box structure for secure group message transmission of CGKA. Then, Hashimoto et al. [35] and Alwen et al. [36] optimize the formal definition to achieve a CGKA protocol with CS support. If a user wants to update the group key, they have to generate the corresponding message and broadcast it to all other users with the help of CS. In large groups, the bandwidth requirements and computation costs are huge due to having users update keys sequentially. Weidner et al. [37] proposed a decentralized CGKA protocol with strong security guarantees, which realizes the linear complexity of communication. Recently, Hashimoto et al. [8] provided a simple and generic wrapper protocol that upgrades non-metadata-hiding CGKAs into metadata-hiding CGKAs. They leverage the existence of a unique continuously evolving group secret key shared among the group members, which is used to convince CS anonymously that a user is a legitimate group member.

Based on the above research, we applied the CGKA protocol to IoV and proposed the ER-CGKA scheme. It aims to ensure a secure message transmission over public channels, protect the privacy of vehicles, and improve the efficiency of the group key updates.

## 3 Preliminary knowledge

### 3.1 Notations

Table 1 shows the main notations and descriptions in the ER-CGKA scheme.

**Table 1:** The main notations and descriptions.

Notations	Descriptions	Notations	Descriptions
*q*	A large prime number	G	An addictive cyclic group with order *q*
*P*	A generator of G	*params*	System’s public parameters
*a*	The private key of TMA	*T*_*pub*_	The public key of TMA
*V*_*i*_	The *i*-th vehicle	*K*_0_	The group key
*x*_*i*_	The private key of *V*_*i*_	*X*_*i*_	The public key of *V*_*i*_
*RID*_*i*_	The real identity of *V*_*i*_	*PID*_*i*,*j*_	The *j*-th pseudonym of *V*_*i*_
$x_{R_{j}}$	The private key of RSU_*j*_	$X_{R_{j}}$	The public key of RSU_*j*_
gsk_*i*_	The group authentication private key of epoch *i*	gvk_*i*_	The group authentication public key of epoch *i*
*p*_*i*_	Proposal generated by *V*_*i*_	*c*_*i*_	Commit corresponding to *p*_*i*_
ch_*i*_	The *i*-th random challenge generated by CS	*σ*_*i*_	The signature of ch_*i*_ generated by vehicles
$\vec{w}$	Welcome message	permKey	The permuted key used to compute index
*TK*_*i*_	The temporary symmetric key	*CT*_*i*_	Ciphertext encrypted with *TK*_*i*_

### 3.2 System model

**Fig 2:**
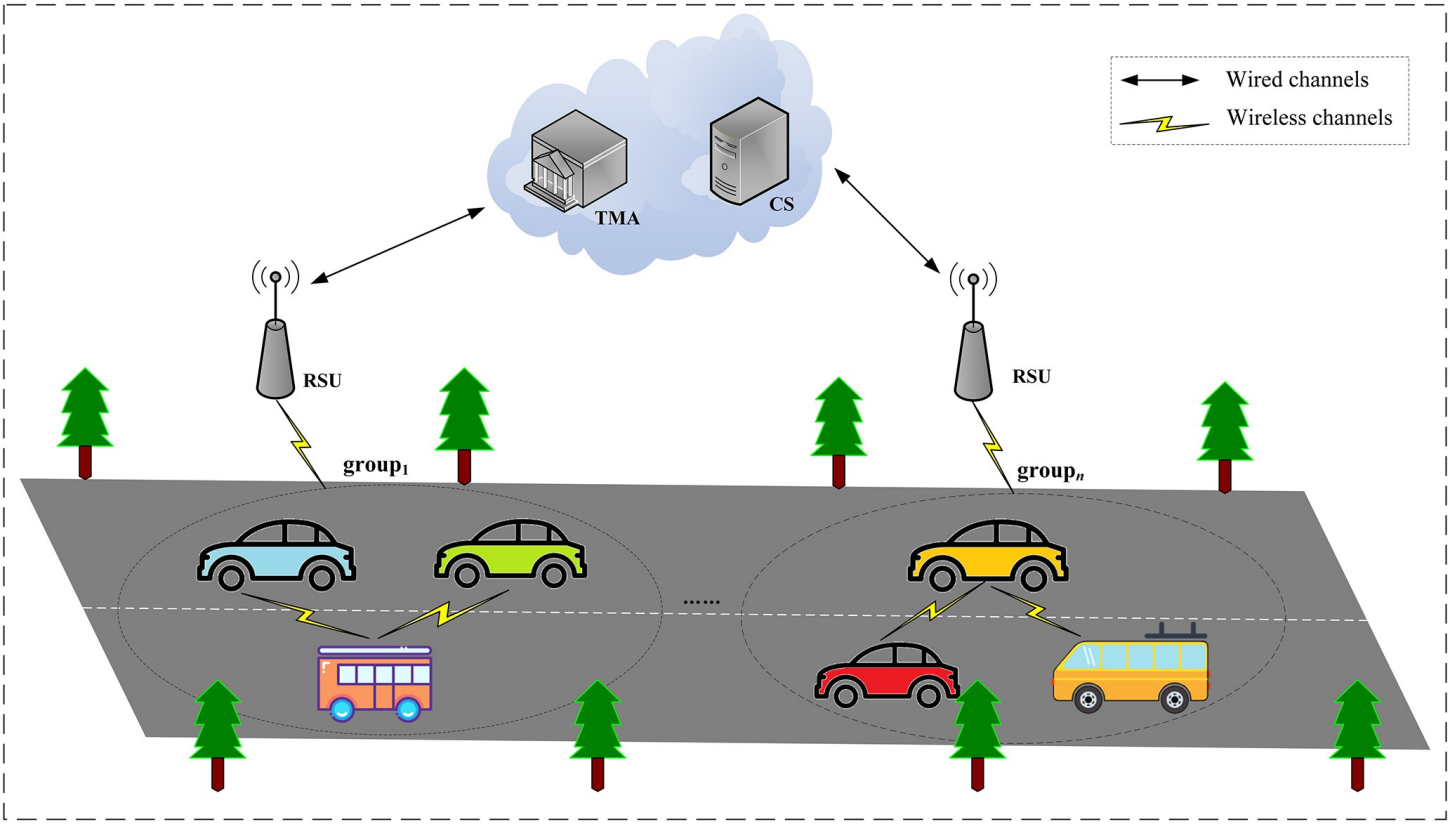
System model of the ER-CGKA scheme.

The system model of our scheme is shown in Fig 2, which includes four entities.

1. **Vehicle.** The vehicle is the main participant in performing group key agreements. It is responsible for creating groups, uploading proposals, generating and processing commits, and updating group keys. Vehicles are incredible since they may be comprised or collude with each other.
2. **RSU.** A semi-trusted RSU is an infrastructure installed along roadsides, which assists vehicles in group key agreements and provides communication transfer services. Although the RSU can perform the protocol honestly, it may record the transmitted data due to curiosity and snoop on the privacy of the vehicle.
3. **TMA.** TMA is a powerful traffic management center. It is responsible for system initialization, pseudonym generation, and key distribution. TMA can retrieve the real identities of malicious vehicles and publish them. TMA is totally credible because it controls the construction and operation of the system.
4. **CS.** CS is the semi-trusted third party that stores the group information, proposals, and commits and informs vehicles to update the group key. Although CS performs the protocol frankly, it tries to snoop on the stored data and identities of vehicles.

### 3.3 Security requirements

1. **Correctness.** All group vehicles have the same group key and state at every same epoch.
2. **Conditional privacy-preserving.** Apart from TMA, the real identities of vehicles are anonymous for other entities.
3. **Confidentiality.** The group key is secret to any entity other than the group members.
4. **Unlinkability.** CS cannot link to two or more proposals or commits that are uploaded or downloaded by the same vehicles.
5. **PCFS.** Although the current group key is disclosed, attackers cannot obtain the previous and subsequent group keys. In other words, the group key at each epoch is incalculable for attackers.
6. **Robustness to collusion attacks.** Suppose there are *n* vehicles in the group, the group key updates only when at least *k* vehicles commit the same proposal.

### 3.4 PRF & PRP

1. **Pseudorandom function (PRF).** Define function F: {0, 1}* × {0, 1}* → {0, 1}*, for adversaries in probability polynomial time (PPT), if $|\text{Pr}[D^{F_{k}\left(.\right)}\left(1^{n}\right)=1]-\text{Pr}[D^{f(.)}(1^{n})=1]|\leq negl(n)$ holds, function F is a PRF. Input key *k* ∈ *K* and binary string *x* ∈ *X*, PRF performs *y* = PRF(*k*, *x*) and outputs *y* ∈ *Y* and *y* is indistinguishable for adversaries in PPT.
2. **Pseudorandom permutation (PRP).** Define function P:{0, 1} * × {0, 1} * → {0, 1}*, for adversaries in PPT, if $|\text{Pr}[D^{F_{k}\left(.\right),F_{k}^{-1}\left(.\right)}\left(1^{n}\right)=1]-\text{Pr}[D^{f(.),f^{-1}(.)}(1^{n})=1]|\leq negl(n)$ holds, function P is a strong PRP.

### 3.5 Syntax of metadata-hidden CGKA

The syntax of metadata-hidden CGKA is given according to [8], where we assume id is the executing party and omit it from the input.

1. Group Creation (Create): Initialize a new group state and take party id as the only member.
2. Proposals (Propose, act)→*p*: Input action ‘act’ which can be ‘add’-id_*t*_ (adding id_*t*_), ‘rem’-id_*t*_ (removing id_*t*_) or ‘upd’ (updating the key material of id_*t*_), output a proposal *p*.
3. Commit (commit,$\vec{p}$)$\to (c_{0},\vec{c},\vec{w})$: Commit a vector of proposals $\vec{p}$, output commit $\left(c_{0},\vec{c}\right)$. *c*_0_ is a member-independent commit, while $\vec{c}={\left({\hat{c}}_{id^{\prime }}\right)}_{id^{\prime }}$ is a member-dependent commit list, where $|\vec{c}|$ is equal to the current group size. If $\vec{p}$ contains a ‘add’ proposal, then it outputs a welcome message $\vec{w}={\left({\hat{w}}_{id_{t}}\right)}_{id_{t}}$, where id_*t*_ is the added member.
4. Process (Process,*c*_0_,${\hat{c}}_{id}$,$\vec{p}$): Process commit $\left(c_{0},{\hat{c}}_{id}\right)$ with the associated proposals $\vec{p}$, move the id’s group state into next epoch.
5. Join (Join,${\hat{w}}_{id}$): The added member id can join group with welcome message ${\hat{w}}_{id}$ and its group state is synced with any member who processes the commit made at the same epoch.
6. Key (Key)→*K*: Output the current group secret key *K*.

## 4 The ER-CGKA scheme

**Fig 3:**
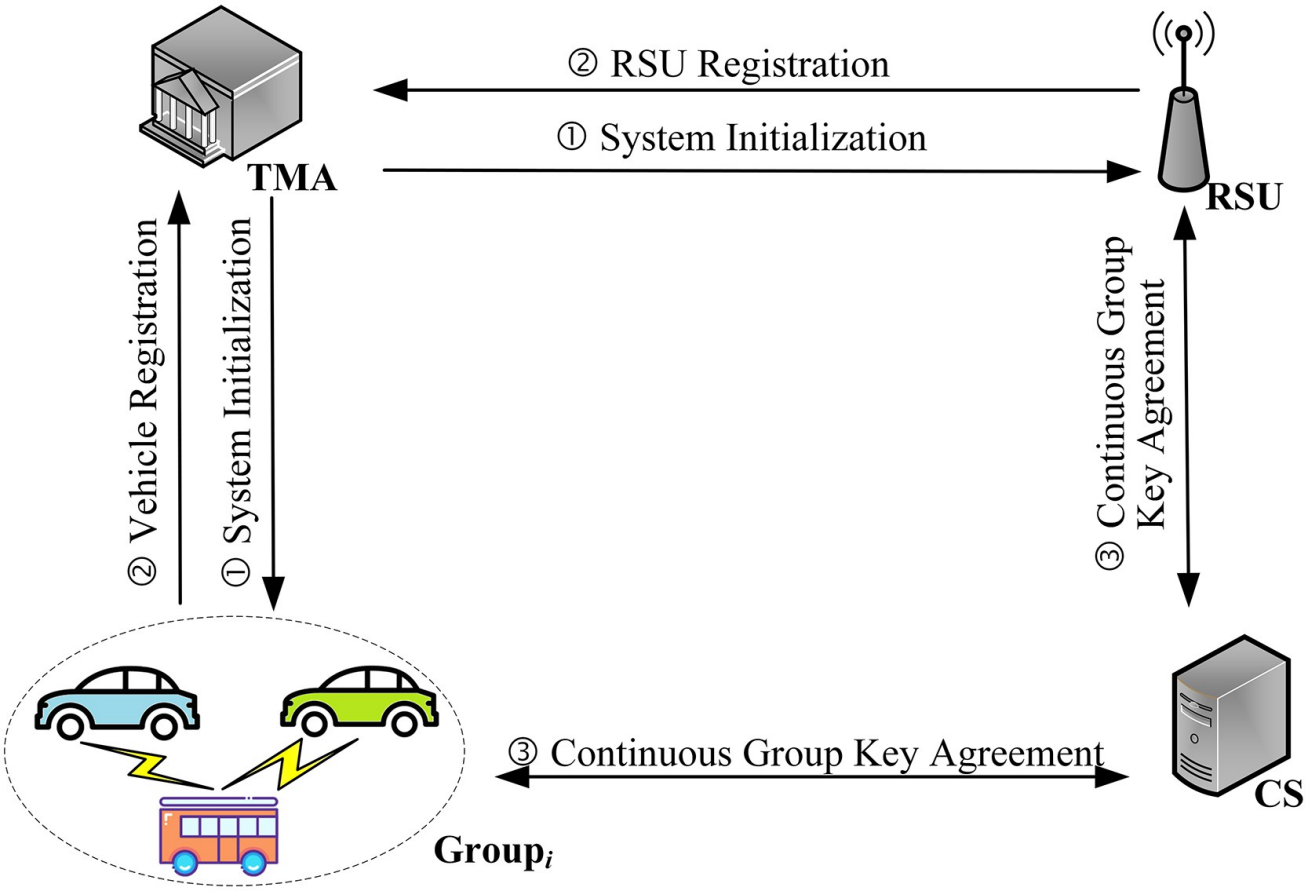
The main procedure of the ER-CGKA scheme.

The main procedure of the ER-CGKA scheme is shown in Fig 3, which contains three parts: **System Initialization**, **Entity Registration** and **CGKA**. Part ① is **System Initialization**, TMA generates its public-private key pairs, and broadcasts system public parameters to RSUs and vehicles. Part ② is **Entity Registration**, TMA generates pseudonyms for vehicles and distributes private keys for RSUs. Part ③ is **CGKA**, which is the core of the ER-CGKA scheme. It includes five stages: group creation, proposals, commit, join, and process & key.

### 4.1 System initialization

At this stage, TMA generates its private key and system public parameters. The steps are as follows.

(1) Input secure parameter λ, choose an additive cyclic group G with order *q*, where *P* is the generator of G.
(2) Randomly choose $a\in Z_{q}^{*}$ as its private key and keep it in secret, set public key *T*_*pub*_ = *aP*.
(3) Choose one secure one-way hash function: $H_{1}:G\times Z_{q}^{*}\to Z_{q}^{*}$.
(4) Publish the system public parameters $params=\{q,P,G,T_{pub},H_{1}\}$.

### 4.2 Entity registration

#### 4.2.1 Vehicle registration

At this stage, vehicle *V*_*i*_ asks TMA for a registration. In the IoV system, a pool of pseudonyms is necessary to protect the identity privacy of the vehicle. The steps are as follows.

(1) *V*_*i*_ randomly chooses $x_{i}\in Z_{\text{q}}^{*}$, computes *X*_*i*_ = *x*_*i*_*P*, transmits registration request {*X*_*i*_, *RID*_*i*_, *n*} to TMA via secure channels, where *n* denotes the number of requested pseudonyms.
(2) After receiving {*X*_*i*_, *RID*_*i*_, *n*}, for *j* = 1, 2, …, *n*, TMA computes *h*_1*i*_ = *H*_1_(*aX*_*i*_||*T*_*i*,*j*_), *Q*_*i*,*j*_ = *RID*_*i*_ ⊕ *h*_1*i*_, *PID*_*i*,*j*_ = {*Q*_*i*,*j*_, *T*_*i*,*j*_}, sets pseudonyms *PID* = {*PID*_*i*,1_, *PID*_*i*,2_, …, *PID*_*i*,*n*_}, and sends *PID* to *V*_*i*_ through secure channels, where *T*_*i*,*j*_ is the valid period of *PID*_*i*,*j*_.
(3) Upon receiving *PID*, *V*_*i*_ stores them to OBU.

#### 4.2.2 RSU registration

At this stage, TMA generate public-private key pairs for RSU_*j*_. The steps are as follows.

(1) RSU_*j*_ transmits its real identity $RID_{R_{j}}$ to the TMA through the secure channel.
(2) TMA randomly chooses $x_{R_{j}}\in Z_{q}^{*}$, computes $X_{R_{j}}=x_{R_{j}}P$, and sends $\left(x_{R_{j}},X_{R_{j}}\right)$ to RSU_*j*_.
(3) RSU_*j*_ keeps $x_{R_{j}}$ in secret and broadcasts $X_{R_{j}}$ to surrounding vehicles.

### 4.3 Continuous group key agreement

In the CGKA protocol, all the vehicles in a group need to store the group identifier gid, the current epoch of the group, the current group key, and information to identify the current members. Additionally, each vehicle stores a public key (called key material) whose corresponding secret key is known only to themselves.

**Fig 4:**
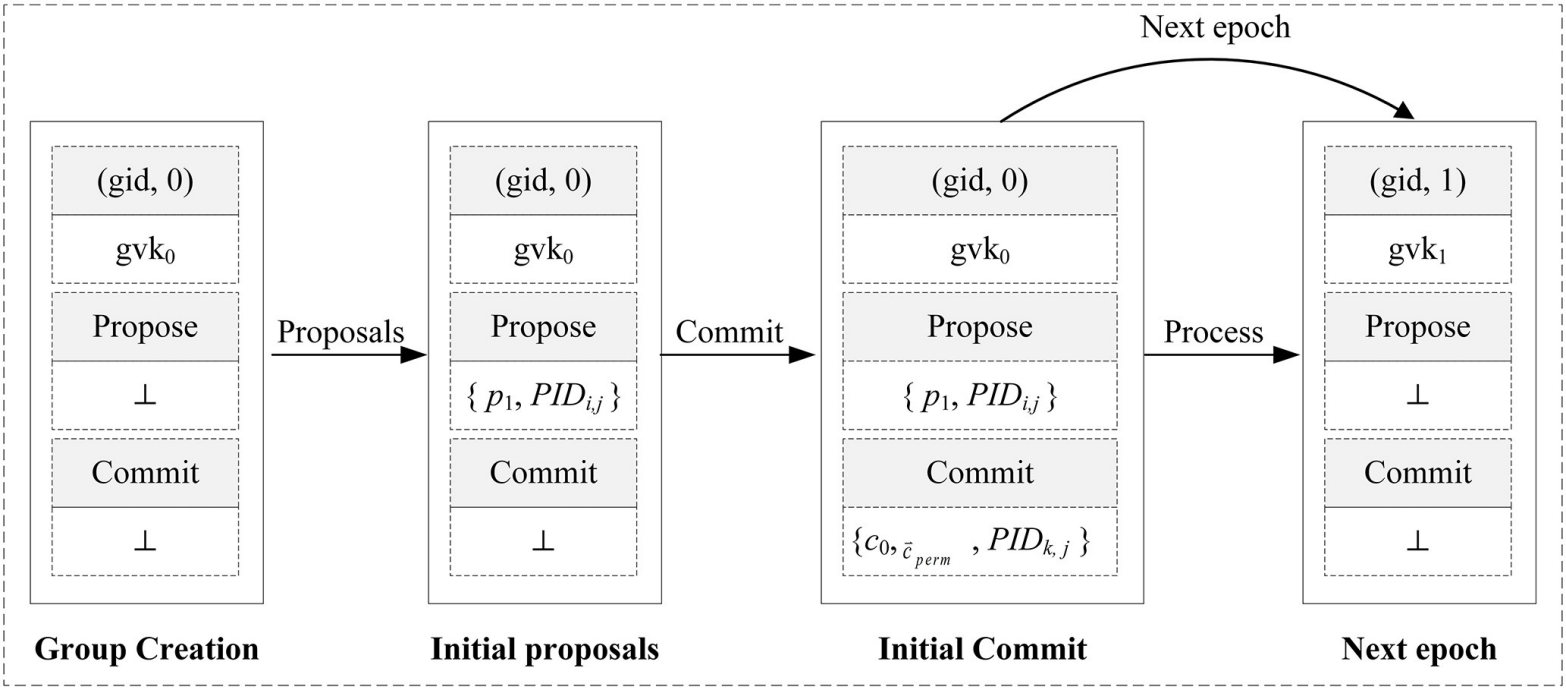
The database (DB) stored on CS.

**Fig 5:**
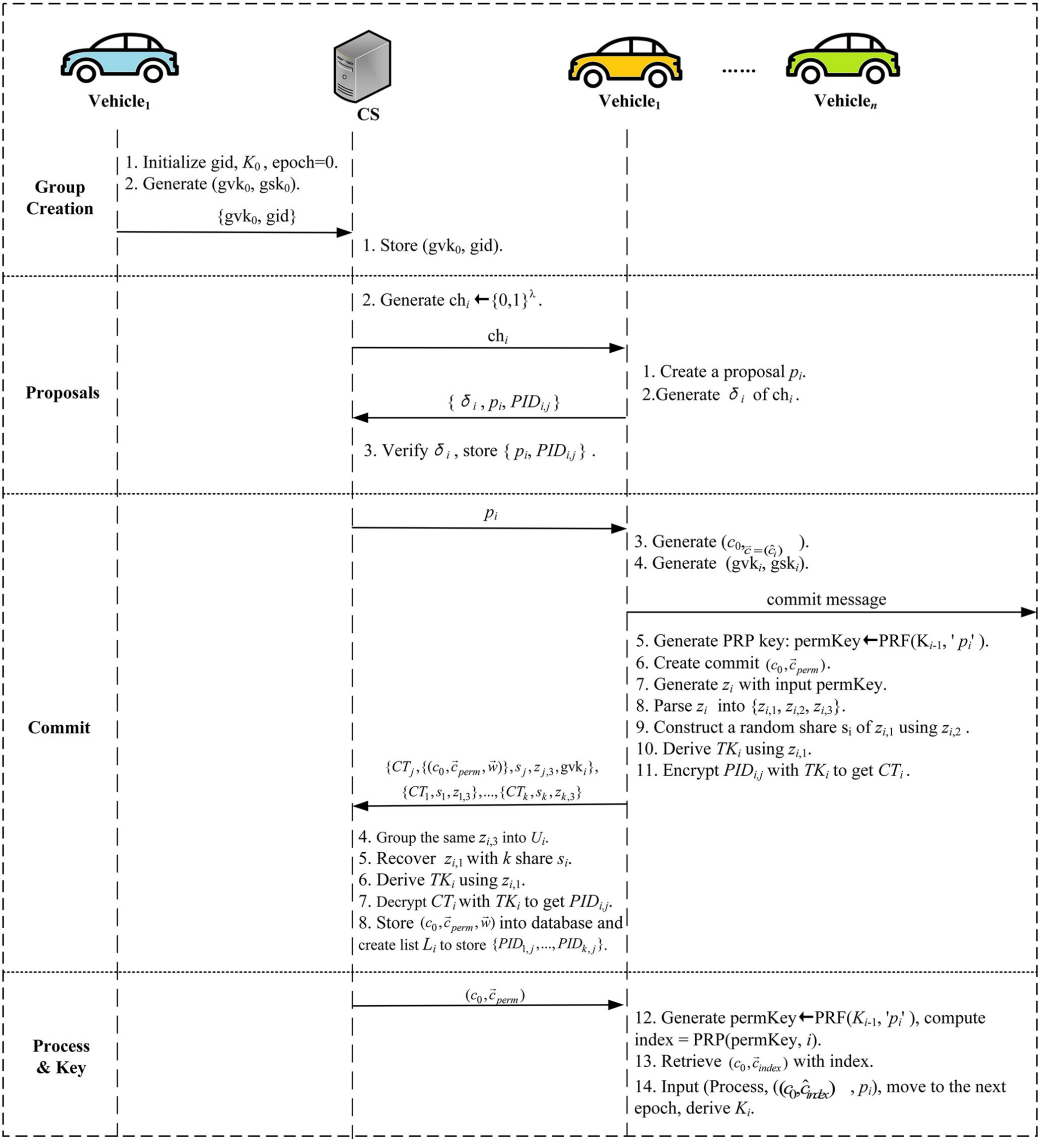
The process of ER-CGKA.

A three-part box representing the group state at a given epoch is stored on CS. As is shown in Fig 4, the top box stores the group identifier gid, epoch, and the group statement gvk_*i*_. The middle box stores the encrypted proposals created during the epoch. The bottom box stores the encrypted commit message that concluded this epoch. Fig 5 shows the process of CGKA in our ER-CGKA scheme.

#### 4.3.1 Group creation

Assume that *V*_1_ hopes to create a group containing *n* members. It first registers a new group that only includes itself to CS. The steps are as follows.

(1) Input (Create), initiate a new group identifier gid and group key *K*_0_, epoch = 0.
(2) Generate group specific authentication key (gvk_0_, gsk_0_) with the group key *K*_0_ and some authentication information.
(3) Send (gvk_0_, gid) to CS.
(4) CS stores (gvk_0_, gid) into the DB.

#### 4.3.2 Proposals

CS generates a random challenge ch_*i*_ ← {0, 1}^λ^ and transmits it to *V*_*i*_ before it uploads the proposals. The steps are as follows.

**Fig 6:**
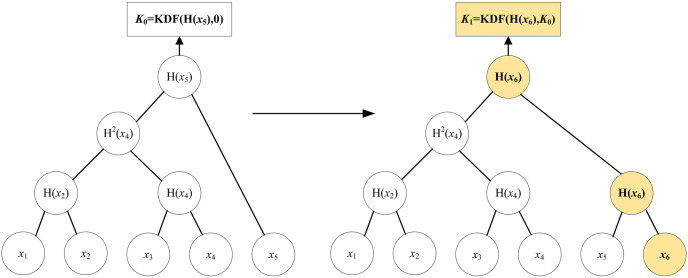
The key agreement for new vehicle joining.

**Fig 7:**
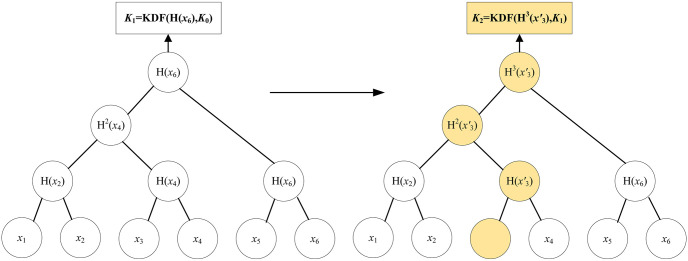
The key agreement for old vehicle leaving.

**Fig 8:**
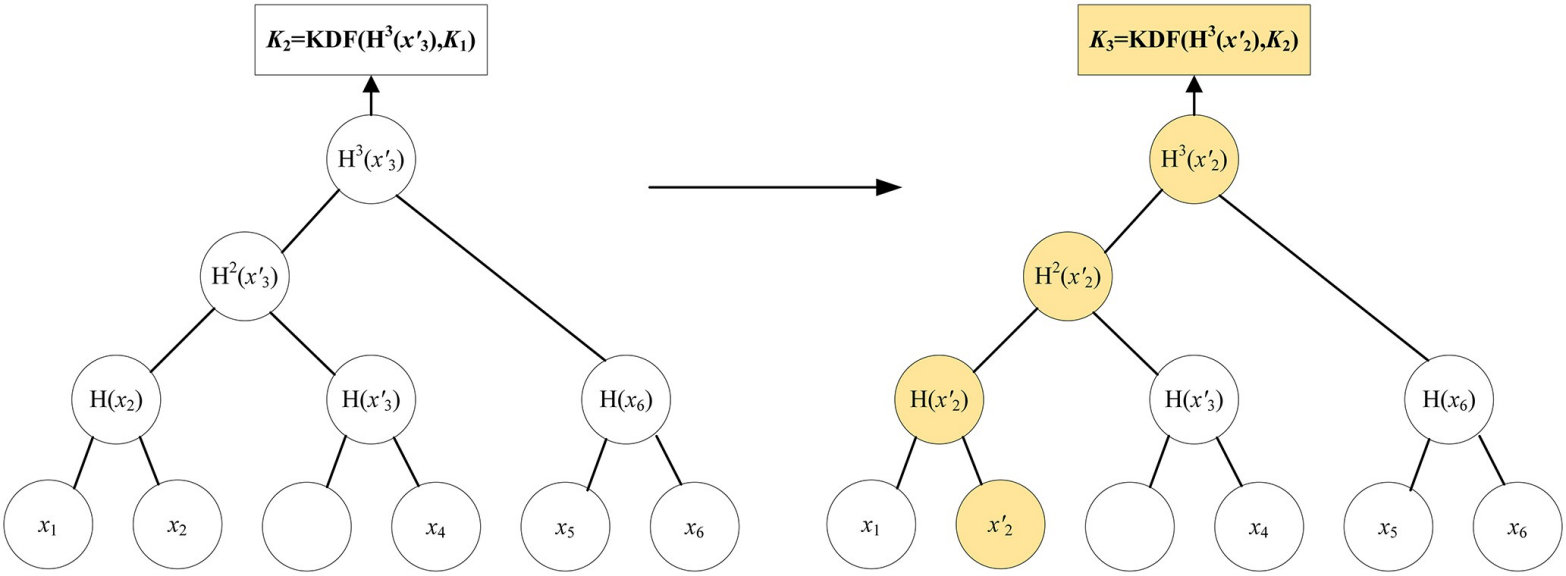
The key agreement for key materials update.

(1) *V*_*i*_ inputs (Propose, ‘act-*PID*_*i*,*j*_’), creates a proposal *p*_*i*_, where ‘act’ ∈ add, rem, upd, represents addition or removal of vehicle *PID*_*i*,*j*_, and update of key materials, respectively.
(2) *V*_*i*_ generates signature *σ*_*i*_ with gsk_*i*_, uploads {*σ*_*i*_, *p*_*i*_, *PID*_1*j*_} to CS.
(3) CS stores {*p*_*i*_, *PID*_*i*,*j*_} into DB after checking the validity of *σ*_*i*_ with gvk_*i*_.

- **Add group member *PID*_*i*,*j*_:** $\left(\gamma^{\prime },p\right)\leftarrow \text{add}\left(\gamma ,PID_{i,j},\text{p}{\text{k}}_{i}\right)$ , propose to add a new vehicle *PID*_*i*,*j*_ to the current group. Input group state, the *PID*_*i*,*j*_ of new vehicle and its public key, output an updated group state and an addition proposal message. Fig 6 shows the key agreement for new vehicles joining. Assume that there are five vehicles in the current group, *V*_1_ proposes to add vehicle *V*_6_ to the group.
- **Remove group member *PID*_*i*,*j*_:** $\left(\gamma^{\prime },p\right)\leftarrow \text{rem}\left(\gamma ,PID_{i,j}\right)$ , propose to remove a vehicle *PID*_*i*,*j*_ from current group. Input group state, the *PID*_*i*,*j*_ of target vehicle, output an updated group state and a removal proposal message. Fig 7 shows the key agreement for old vehicles leaving. Assume that *V*_1_ proposes to remove vehicle *V*_3_ from the group.
- **Update key material:** $\left(\gamma^{\prime },p\right)\leftarrow \text{upd}\left(\gamma \right)$ , propose to update the key materials of vehicles. Input group state, output an updated group state and an update proposal message. Fig 8 shows the group key agreement for the key materials update. Assume that *V*_2_ updates its new key as $V_{2}^{\prime }$.

#### 4.3.3 Commit

To update the group state, *V*_*i*_(*i* = 1, 2, …, *n*) download *p*_*i*_ to commit. The steps are as follows.

1. **Initial commit phase.** Vehicle *V*_*j*_ performs the following algorithms.
  (1) Input (Commit, *p*_*i*_), generate a commit $\left(c_{0},\vec{c}={\left({\hat{c}}_{i}\right)}_{i\in [1,2,...,n]},\vec{w}\right)$. The welcome message $\vec{w}={\left({\vec{w}}_{\text{i}{\text{d}}_{i}}\right)}_{\text{i}{\text{d}}_{i}}$ is depended on if there is an additional proposal message in $\vec{p}$.
  (2) Generate new group authentication key (gvk_*i*_, gsk_*i*_).
  (3) Inform vehicles that it has generated the commit and gvk_*i*_.
  (4) Generate PRP key $\text{permKey}\leftarrow \text{PRF}(K_{i-1}{,}^{\prime }{p_{i}}^{\prime })$, which defined the permutation on group size.
  (5) Create a permuted member dependent commit ${\vec{c}}_{perm}$ so that ${\hat{c}}_{i}$ is placed at entry PRP(permKey, *i*) ∈ [1, 2, …, *n*].
2. **Message construction phase.** Vehicles *V*_*i*_(*i* = 1, 2, …, *n*) perform the following algorithms.
  (1) Execute step (4) in **Initial commit phase** except *V*_*j*_.
  (2) Operate the VOPRF protocol [38], input permKey, output *z*_*i*_.
  (3) Parse *z*_*i*_ into three parts (*z*_*i*,1_, *z*_*i*,2_, *z*_*i*,3_) ∈ {0, 1}^λ^ × {0, 1}^λ^ × {0, 1}^λ^.
  (4) Construct a random share *s*_*i*_ of *z*_*i*,1_ using a (*k*, *n*) secret-sharing scheme [39], where *z*_*i*,2_ is used in the share generation process.
  (5) Derive a temporary symmetry key *TK*_*i*_ using a pseudorandom generator, where takes *z*_*i*,1_ as input.
  (6) Encrypt *PID*_*i*,*j*_ with *TK*_*i*_ to obtain cyphertext *CT*_*i*_.
  (7) *V*_*j*_ uploads $\left\{CT_{j},\left\{\left(c_{0},{\vec{c}}_{perm},\vec{w}\right)\right\},s_{j},z_{j,3},\text{gv}{\text{k}}_{i}\right\}$ to CS after performing an authentication with gsk_*i*_, while other vehicles uploads {*CT*_*i*_, *s*_*i*_, *z*_*i*,3_}.
3. **Aggregation phase.** CS performs the following algorithms.
  (1) Group together messages that share the same *z*_*i*,3_ vale into subset *U*_*i*_.
  (2) Run the share recovery algorithm on the collections of share values {*s*_*i*_}_*i* = 1, …*k*_ ∈ *U*_*i*_ to output *z*_*i*,1_.
  (3) Derive a temporary symmetry key *TK*_*i*_ takes *z*_*i*,1_ as input.
  (4) Decrypt *CT*_*i*_ with *TK*_*i*_ to obtain *PID*_*i*,*j*_.
  (5) Store $\left(c_{0},{\vec{c}}_{perm},\vec{w}\right)$ into DB and create list *L*_*i*_ to store {*PID*_1,*j*_, *PID*_2,*j*_, …, *PID*_*k*,*j*_}.

#### 4.3.4 Join

It is worth noting that the added vehicle *PID*_*i*,*j*_ can join in group using welcome message $w_{PID_{i,j}}$ if ‘act’ = add.

(1) After *V*_1_ uploading $\left(c_{0},{\vec{c}}_{perm},\vec{w}\right)$, CS stores $\vec{w}$ into a specific DB, where works similarly to the $F_{\text{CGKA}}^{\text{ctxt}}$.
(2) *V*_*i*_(*i* = 1, 2, …, *n*) performs authentication protocol to retrieve $w_{PID_{i,j}}$ in the DB of CS. Input (Join, $w_{PID_{i,j}}$), the state of *PID*_*i*,*j*_ is synced with any member who processes the commit made at the same epoch.

#### 4.3.5 Process & Key

Once there are *k* elements in *L*_*i*_, CS broadcasts $\left(c_{0},{\vec{c}}_{perm}\right)$ to all vehicles. Then, *V*_*i*_(*i* = 1, 2, …, *n*) retrieves and processes commit corresponding to themselves. Steps are as follows.

(1) Generate permKey = PRF (*K*_*i*−1_,′ *p*_*i*_′), compute the permuted index = PRP (permKey, *i*).
(2) Retrieve $\left(c_{0},{\vec{c}}_{index}\right)$ from $\left(c_{0},{\vec{c}}_{perm}\right)$ with index.
(3) Input $\left(\text{Process},\left(c_{0},{\hat{c}}_{\text{index}}\right),p_{i}\right)$, move to the next epoch and generate the new group key *K*_*i*_.

## 5 Security proof and security analysis

### 5.1 Security proof

The priority of all CGKA schemes is to prevent the adversary from getting the group key. We prove the ER-CGKA scheme is secure according to the CGKA security model in [40].

In the CGKA security game, the attacker is given access to various oracles to drive the execution of a CGKA protocol. However, the attacker will not be allowed to modify or inject any control messages. In addition, the capabilities of the attacker and restrictions on the order in which the attacker may call the oracles are motivated by a CGKA protocol, which would be used at a higher level. The main oracles to drive the execution are the oracles **‘create-group’**, **‘add-user’**, **‘remove-user’**, **‘send-update’**, **‘deliver’**. The first four oracles allow the adversary to instruct vehicles to initiate new epochs, whereas the deliver oracle makes vehicles proceed to the next epoch. CS connecting the vehicles is trusted to provide vehicles with a consistent view of which operation takes place in each epoch. That is, while multiple parties may initiate a new epoch, the attacker is forced to pick a single operation that defines the new epoch; the corresponding sender is referred to as the leader of the epoch.

The game forces the attacker to initially create a group at epoch = 0. Thereafter, any group member may add new vehicles, remove current group members, or perform an update. The attacker may also corrupt any vehicle at any point and challenge the update secret in any epoch where the leader performs an update operation. Furthermore, the adversary can instruct vehicles to stop deleting old secrets. There will be restrictions checked at the end of the execution of the game to ensure that the attacker’s challenge/corruption/no-deletion behavior does not lead to trivial attacks.

Then, we prove the ER-CGKA scheme is secure through a game interaction between adversary *A* and oracles.

**Definition 1 (Non-adaptive CGKA security)** *The CGKA protocol is non-adaptively* (*t*, *c*, *n*, *P*, *ε*)-secure if for all (*t*, *c*, *n*) − *adversaries*, $Adv_{cgka-na}^{CGKA,P}\left(A\;\right)\leq \;\varepsilon$. *In other words, if the adversary* *A* *that runs in time at most* *t*, *makes at most c challenge queries, and never produces a group with more than n members, then the advantage of A with safety predicate P against a CGKA scheme is ε*.

**Theorem 1** *If A can win CGKA security game with the non-negligible probability ε in PPT under the ROM, then A has the advantage of* ${Adv}_{cgka-na}^{CGKA,P}\left(A\;\right)\leq \;\left|\varepsilon -\frac{1}{2}\right|$ *breaking CGKA protocol*.

**Proof 1** *Assumed that A tries to obtain group key, the interaction between A and oracles are as follows*.

- ***Initialization*.** *The init oracle sets up the game and all the variables need to keep track of the execution. The random bit b is used for real-or-random challenges, and the dictionary γ keeps track of all the vehicles’ states. For every epoch, the dictionaries lead, I, and G record the leader, the update secret, and the group members, respectively, and ep records which epoch each vehicle is currently in. The array ctr counts all new operations initiated by a vehicle in its current epoch. Moreover, D keeps track of which parties delete their old values and which do not. Dictionary chall is used to ensure that the adversary A can issue at most a single challenge per (update) epoch. Finally, M records all control messages produced by parties; the adversary A has read access to M (as indicated by the keyword pub)*.
- ***Initiating operations and choosing epoch leaders*.** *As mentioned above, the adversary A must choose a leader in every epoch, i.e., a sender whose control message is ultimately processed by all group members. More precisely, for each vehicle PID*_*i*,*j*_ *currently in some epoch t, ctr*[*PID*_*i*,*j*_] *can be thought of as a (local) “version number” that counts the various operations initiated by PID*_*i*,*j*_ *in epoch t. The counter is incremented each time PID*_*i*,*j*_ *initiates a new operation. The resulting control messages for vehicles PID*_*i*,*j*_ *are stored in M with key* (*t* + 1, *PID*_*i*,*j*_, *PID*_*k*,*j*_, *ctr*[*PID*_*i*,*j*_]), *representing the number of the next epoch, the sender, the recipient, and the (local) version number of the operation. Similarly, dictionary G stores the new group that would result from the operation with key* (*t* + 1, *PID*_*i*,*j*_, *ctr*[*PID*_*i*,*j*_]).
  *For every epoch t, the first control message M*[*t*, *PID*_*i*,*j*_, *PID*_*m*,*j*_, *c*] *delivered via deliver, for some vehicles PID*_*i*,*j*_, *PID*_*m*,*j*_ *and version number c, determines that PID*_*i*,*j*_ *is the leader and c is the version that was chosen by CS. Correspondingly, the game records* (*PID*_*i*,*j*_, *c*) → *lead*[*t*] *and sets the group membership to G*[*t*, *PID*_*i*,*j*_, *c*] → *G*[*t*].
  *In general, whenever a vehicle PID*_*m*,*j*_ *processes any control message, the counter ctr*[*PID*_*m*,*j*_] *is reset to 0 as all operations initiated by PID*_*m*,*j*_ *in its current epoch are now obsolete (either processed by PID*_*i*,*j*_ *or rejected by the server in favor of some other operation). Note that the sender of an operation also sends a control message addressed to themselves to the server. The server confirms an operation by returning that message back to the sender*.
- ***Group creation*.** *The oracle create-group causes* *PID*_1,*j*_ *to create a group with members* {*PID*_1,*j*_, *PID*_2,*j*_, …, *PID*_*n*,*j*_}. *This is only allowed if* *PID*_1,*j*_ *is currently in epoch 0, which is enforced by the req statement. Thereafter*, *PID*_1,*j*_ *calls the group creation algorithm and sends the resulting welcome messages to all vehicles involved (including itself)*.
- ***Adding and removing vehicles and performing updates*.** *For all three oracles add-user, remove-user, and send-update, the req statement checks that the call makes sense (e.g., checking that a vehicle added to the group is not currently a group member). Subsequently, the oracles call the corresponding CGKA algorithms (add, rem, and upd, respectively) and store the resulting control messages in M*.
- ***Delivering control messages*.** *The oracle deliver is called with the same four arguments* [*t*, *PID*_*i*,*j*_, *PID*_*m*,*j*_, *c*] *that are used as keys for the M array. The req statement at the beginning checks that (1) either there is no leader for epoch t yet or version c of PID*_*i*,*j*_ *is the leader already and (2) the recipient PID*_*k*,*j*_ *is currently either in epoch t* − 1 *or a newly added group member, which is checked by predicate added defined by added*[*t*, *PID*_*i*,*j*_, *PID*_*m*,*j*_, *c*] ≔ *PID*_*m*,*j*_ ∉ *G*[*t* − 1] ∧ *PID*_*m*,*j*_ ∈ *G*[*t*, *PID*_*i*,*j*_, *c*]. *If the checks are passed, the appropriate control message is retrieved from M and run through proc on the state of* *PID*_*i*,*j*_. *If there is no leader for epoch* *t* *yet, the game sets the leader as explained above and also records the update secret I*[*t*] *output by proc. In all future calls to deliver, the values* *I output by process will be checked against I*[*t*] *and, in case of a mismatch, the instruction win reveals the secret bit b to the attacker; this ensures correctness. Finally, the epoch counter for PID*_*k*,*j*_ *is incremented—or set to -1 if the operation just processed removes* *PID*_*k*,*j*_ *from the group. This involves a check via predicate removed defined by removed*[*t*, *PID*_*m*,*j*_] ≔ *PID*_*m*,*j*_ ∉ *G*[*t* − 1] ∧ *PID*_*m*,*j*_ ∈ *G*[*t*].
- ***Challenges, corruptions, and deletions*.** *In order to capture that update secrets must look random, the attacker is allowed to issue a challenge for any epoch corresponding to an update operation. When calling chall*[*t*] *for some t, the oracle first checks that t indeed corresponds to an update epoch and that a leader already exists. Similarly, using reveal, the attacker can simply learn the update secret of an epoch. It is also ensured that for each epoch, the attacker can make at most one call to either chall or reveal*.
  *To formally model forward secrecy and PCS, the attacker is also allowed to learn the current state of any vehicle by calling the oracle corrupt. Finally, the attacker can instruct a vehicle PID*_*i*,*j*_ *to stop deleting old values by calling no-del* (*PID*_*i*,*j*_). *Subsequently, the game will implicitly store all old states of PID*_*i*,*j*_ *(instead of overriding them) and leak it to the attacker when he calls corrupt. The game also sets the corresponding flag*.
- ***Avoiding trivial attacks*.** *In order to ensure that the attacker may not win the CGKA security game with trivial attacks (such as, e.g., challenging an epoch t’s update secret and leaking some vehicle’s state in epoch t), at the end of the game, the predicate safe is run on the queries q*_1_, *q*_2_, …, *q*_*q*_ in order to determine whether the execution was devoid of such attacks. The predicate tests whether the attacker can trivially compute the update secret in a challenge epoch t* using the state of a vehicle PID_*i*,*j*_ *in some t and the control messages observed on the network. This is the case if either (1) PID*_*i*,*j*_ has not performed an update or been removed before epoch t* or (2) PID_*i*,*j*_ stopped deleting values at some point up to epoch t* and was corrupted thereafter. It uses the function q2e(*q*), *which returns the epoch corresponding to query q. Specifically, for q* ∈ {*corrupt*(*PID*_*i*,*j*_), *no-del*(*PID*_*i*,*j*_)}, *if PID*_*i*,*j*_ *is member of the group when* **q** *is made, q2e*(*q*) *is the value of ep*[*PID*_*i*,*j*_], *otherwise, q2e*(*q*) *returns* ⊥. *For q* ∈ {*send-update*(*PID*_*i*,*j*_), *remove-user* (*PID*_*i*,*j*_, *PID*_*k*,*j*_)}, *q2e*(*q*) *is the epoch for which* *PID*_*i*,*j*_ *initiates the operation. If q is not processed by any vehicle we set q2e*(*q*) = ⊥. *Observe that the predicate safe can in general be replaced by any other predicate P, potentially weakening the resulting security notion*.
- ***Advantage*.** *Next, we analyze the probability that A can win CGKA security game. A* (*t*, *c*, *n*) − *adversary is an adversary A that runs in time at most t, makes at most c challenge queries, and never produces a group with more than n members. For adversary A for which the safety predicate evaluates to true on the queries made by it, A wins the CGKA security game if it correctly guesses the random bit b in the end. The advantage of A with safety predicate P against a CGKA scheme CGKA is defined by* ${Adv}_{cgka-na}^{CGKA,P}\left(A\right)≔\left|\text{Pr}\left[A\;\text{wins}\right]\text{-}\frac{1}{2}\right|$.

*To sum up, the probability that A can win CGKA security game is* ${Adv}_{cgka-na}^{CGKA,P}\left(A\right)\leq \;|\varepsilon -\frac{1}{2}|$ .

### 5.2 Security analysis

We prove that our scheme satisfies all the security requirements mentioned in Section 3.3.

1. **Correctness.** All vehicles in the same group will compute a new group key and move to the next epoch after processing the same proposal. In addition, if a vehicle joins the group via a welcome message $w_{\text{i}{\text{d}}_{i}}$, its group state is synced with any member who processes the commit made at the same epoch. According to **Theorem 1**, the ER-CGKA scheme contents correctness.
2. **Conditional privacy-preserving**.
  (1) Anonymity. We employ pseudonym technology to protect the identity privacy of vehicles, which makes it impossible for any entity to learn the real identity of vehicles except TMA.
  (2) Traceability. If vehicles send malicious or fake messages, other vehicles will transmit their pseudonyms (e.g. *PID*_*i*,*j*_) to TMA with the help of RSU. Then, TMA retrieves *Q*_*i*,*j*_ and *T*_*i*,*j*_ in its DB with *PID*_*i*,*j*_, computes *h*_1*i*_ = *H*_1_(*aX*_*i*_||*T*_*i*,*j*_) with *a*, recovers the real identity of malicious vehicle *RID*_*i*_ = *Q*_*i*,*j*_⊕*h*_1*i*_, removes it from the group and punishes it.
  Therefore, the ER-CGKA scheme realizes conditional privacy-preserving.
3. **Confidentiality.** The group key is derived from the previous group key and the update key material. So even if an attacker steals the group key of the previous epoch, there is no way to generate the current group key without update key material. In other words, the group key is kept secret from any entity other than the group members. Therefore, the ER-CGKA scheme ensures group key confidentiality.
4. **Unlinkability.** At the vehicle registration stage, TMA generates a batch of pseudonyms so that vehicles can choose one unused pseudonym at a time to communicate with others. Thus, any third party (e.g. CS) cannot link to two or more proposals or commits are uploaded or downloaded by the same vehicle. Therefore, the ER-CGKA scheme fulfills unlinkability.
5. **PCFS.**
  (1) FS. The FS means that the new vehicle joining the group cannot decrypt the communication content of the previous epoch. In our scheme, the group key is commonly derived from the group key at the previous epoch and the key material. The group state of the new vehicle is synchronized with the members at the current epoch, but it cannot obtain the previous group key to decrypt the former ciphertext. According to **Theorem 1**, the ER-CGKA scheme satisfies FS.
  (2) PCS. The PCS means that vehicles that left the group cannot obtain the group key of the next epoch. In our scheme, if a vehicle uploads a ‘rem’ proposal, the group key will be updated once at least *k* vehicles commit it. Since the left vehicle does not know the key materials, it cannot compute the new group key of subsequent epochs. According to **Theorem 1**, the ER-CGKA scheme achieves PCS.
6. **Resistance to collusion attacks.** At the commit stage, vehicles in the group can download proposals to commit and upload the commits to CS. Especially, CS checks if there are at least *k* commits in a *n*-size group that are associated with the same proposal. If so, CS informs vehicles to download the commits to process and update the group key. Therefore, the ER-CGKA scheme can resist collusion attacks through the (*k*, *n*) secret sharing scheme.

Table 2 shows the security comparison result between our scheme and schemes in [8, 13, 14], where “✓” represents satisfying the property, “×” represents not satisfied.

**Table 2:** Security comparison.

Schemes	Scheme [8]	Scheme [13]	Scheme [14]	Ours
Correctness	✓	✓	✓	✓
Conditional privacy-preserving	✓	✓	✓	✓
Confidentiality	✓	✓	✓	✓
Unlinkability	✓	×	×	✓
PCFS	✓	×	×	✓
Resistance to collusion attacks	×	×	×	✓

The scheme in [13] assumed that there is a fully trusted cluster head vehicle, which breaks the secure communication if the cluster head vehicle is compromised. Moreover, the scheme does not enable PCS since the key updates are not considered. The scheme in [14] considers the key update. Nevertheless, the vehicle that has left the group can still compute the updated key by performing the “XOR” operation on the updated key and the old key. Besides, the vehicles in the schemes in [13, 14] use a specific identity for communication, which cannot satisfy the unlinkability. Finally, the schemes in [8, 13, 14] cannot resist collusion attacks, but our ER-CGKA scheme satisfies all the above security requirements.

## 6 Performance evaluation

We analyze the computation and communication overhead of the ER-CGKA scheme. The cost of the scheme in [8] is written in normal font (X), while ours is written in bold (±X). In addition, we simulate the performance of our ER-CGKA scheme. The CGKA protocol is mainly interacted with vehicles and CS. Therefore, the CS is simulated by Windows 10 laptop 607 with an AMD Ryzen 9 5950 X @4 GHz processor and 32 GB of memory. The vehicle (Client) is simulated by Windows 10 laptop with Intel(R) Core (TM) i5-7200 U CPU @ 2.50 GHz and 12 GB of memory.

### 6.1 Computation cost

For computation cost, we consider that (1) The run time of key generation like signature key *T*_*gk*_, group key *T*_*k*_, symmetric key *T*_*der*_ and permKey *T*_*PRP*_. (2) The time of executing algorithms like VOPRF protocol *T*_*VOPRF*_, secret sharing *T*_*ss*_ and recovery *T*_*rec*_. (3) The general run time like signature generation *T*_*sig*_ and verification *T*_*ver*_, symmetric encryption *T*_*se*_ and symmetric decryption *T*_*sd*_. The details are described as Table 3.

**Table 3:** The comparison of computation cost.

Procedure	Client	CS
Group Creation	*T*_*gk*_+*T*_*k*_	
Proposals	*T*_*sig*_	*T*_*ver*_
Commit	*T*_*gk*_+*T*_*PRP*_+*T*_*sig*_ (+***T*_*ss*_**+***T*_*VOPRF*_**+***T*_*der*_**+***T*_*se*_**)	*T*_*ver*_ (+***T*_*rec*_**+***T*_*der*_**+***T*_*sd*_**)
Process & Key	*T*_*sig*_+*T*_*PRP*_+*T*_*k*_ (-***T*_*sig*_**)	*T*_*ver*_ (-***T*_*ver*_**)

According to Table 3, the cost of the ER-CGKA scheme is equal to the scheme in [8] during **Group Creation** and **Proposals**. In the **Commit** stage, our scheme takes more time due to resisting collusion attacks. Concretely, the extra time is executing VOPRF protocol once, performing a secret share and recovery scheme, deriving the symmetric key twice, and operating a pair of symmetric encryption and decryption. In the **Process & Key** stage, CS broadcasts commit messages to vehicles rather than download from CS. Thus, we do not need to perform an authentication protocol.

**Fig 9:**
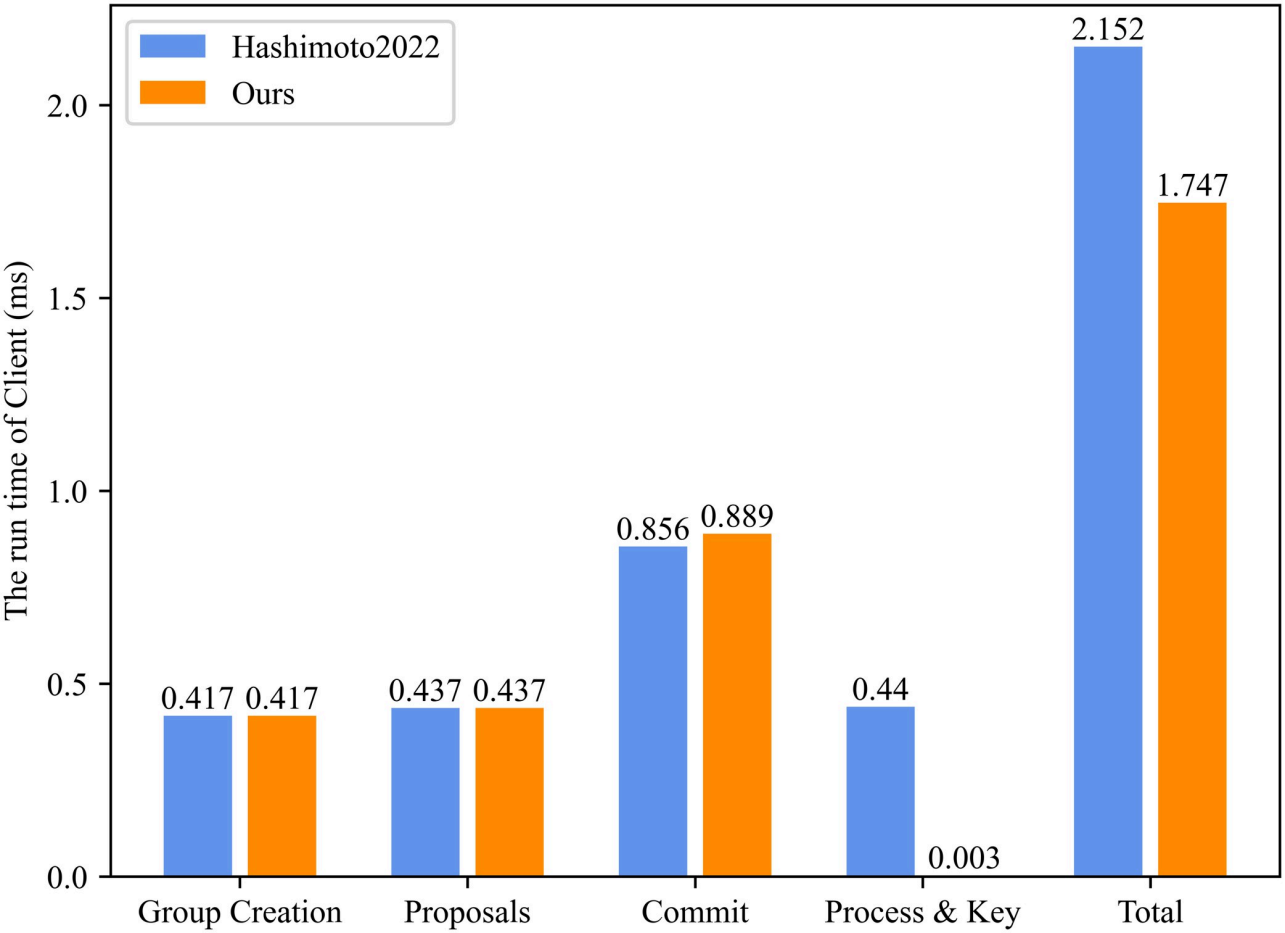
The run time of client.

**Fig 10:**
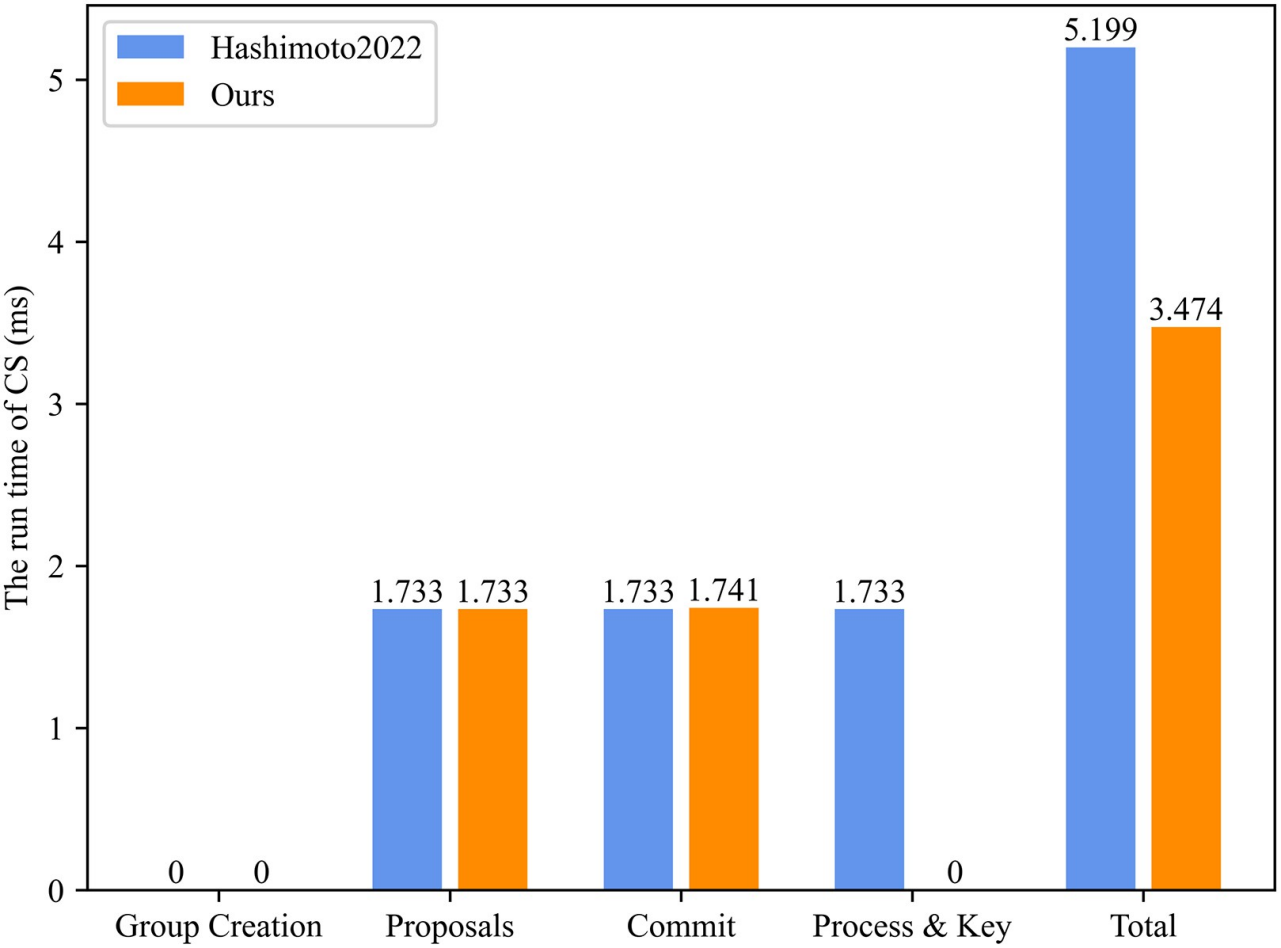
The run time of CS.

Based on Table 3, we simulated the running time of Client and CS in Figs 9 and 10. Specifically, the signature algorithm is the elliptic curve digital signature algorithm (ECDSA), and the symmetric encryption algorithm is the advanced encryption standard (AES). According to Fig 9, we can see that at the stages of Group Creation (0.417 ms) and Proposals (0.437 ms), the run time of the Client in our proposed ER-CGKA scheme is equal to the scheme in [8]. During the Commit stage, our run time (0.889 ms) is a little higher than the scheme in [8] (0.856 ms) since the secret sharing the algorithm is executed to resist the collusion attacks by the group members. At the Process & Key stage, compared to the the scheme in [8] (0.44 ms), the computation time in proposed ER-CGKA scheme (0.003 ms) has reduced 99.32%. Overall, our the total computation time of the Client has decreased by 18.82%. Similarly, according to Fig 10, compared to the scheme in [8], the run time of CS in our proposed ER-CGKA scheme at the Process & Key stage has reduced 100%, and the total time decreased by 33.18%. Therefore, our ER-CGKA scheme is more efficient.

### 6.2 Communication overhead

In terms of communication overhead, we consider the bandwidth requirements of pseudonym |*PID*_*i*,*j*_|, group key |ek|, commit message |*c*_0_|, |${\hat{c}}_{id}$|, signature |sig|, and signature authentication key |svk|. U (resp. A, R) stand for the number of ‘upd’ (resp. ‘add’, ‘rem’) proposals published during the last epoch. The details are described as Table 4.

**Table 4:** The comparison of communication overhead.

Procedure	Upload						Download				
	|*PID*_*i*,*j*_|	|ek|	|*c*_0_|	|${\hat{c}}_{id}$|	|sig|	|svk|	|ek|	|*c*_0_|	|${\hat{c}}_{id}$|	|sig|	|svk|
Propose—‘upd’	**(+1)**	1			3						
Propose—‘add’	**(+1)**	1			2	1					
Propose—‘rem’	**(+1)**				2						
Commit	(+*k*)	1	1	N	4	1	U+A			2U+A+R	A
Process & Key					1**(-1)**		U+A+1	1**(-1)**	1**(-1)**	2U+A+R+2	A

As is shown in Table 4, the bandwidth overhead is added on uploading the pseudonym of the vehicle in **Propose** and **Commit** stage, which is used to trace the real identity of the proposer for TMA and realize resistance to collusion attacks. Besides, the cost is reduced in the **Process & Key** stage. On the one hand, vehicles do not need to upload signatures to convince CS that it is a legitimate group member. On the other hand, the commit message downloading is not required since CS broadcasts to them directly.

We applied ElGamal mPKE and ECDSA algorithms to obtain concrete communication overhead. We assume a group of *N* = 256 members, *k* = 2/3*N*=171 members, and numbers for Commit and Process assume no proposal was made during the last epoch.

**Fig 11:**
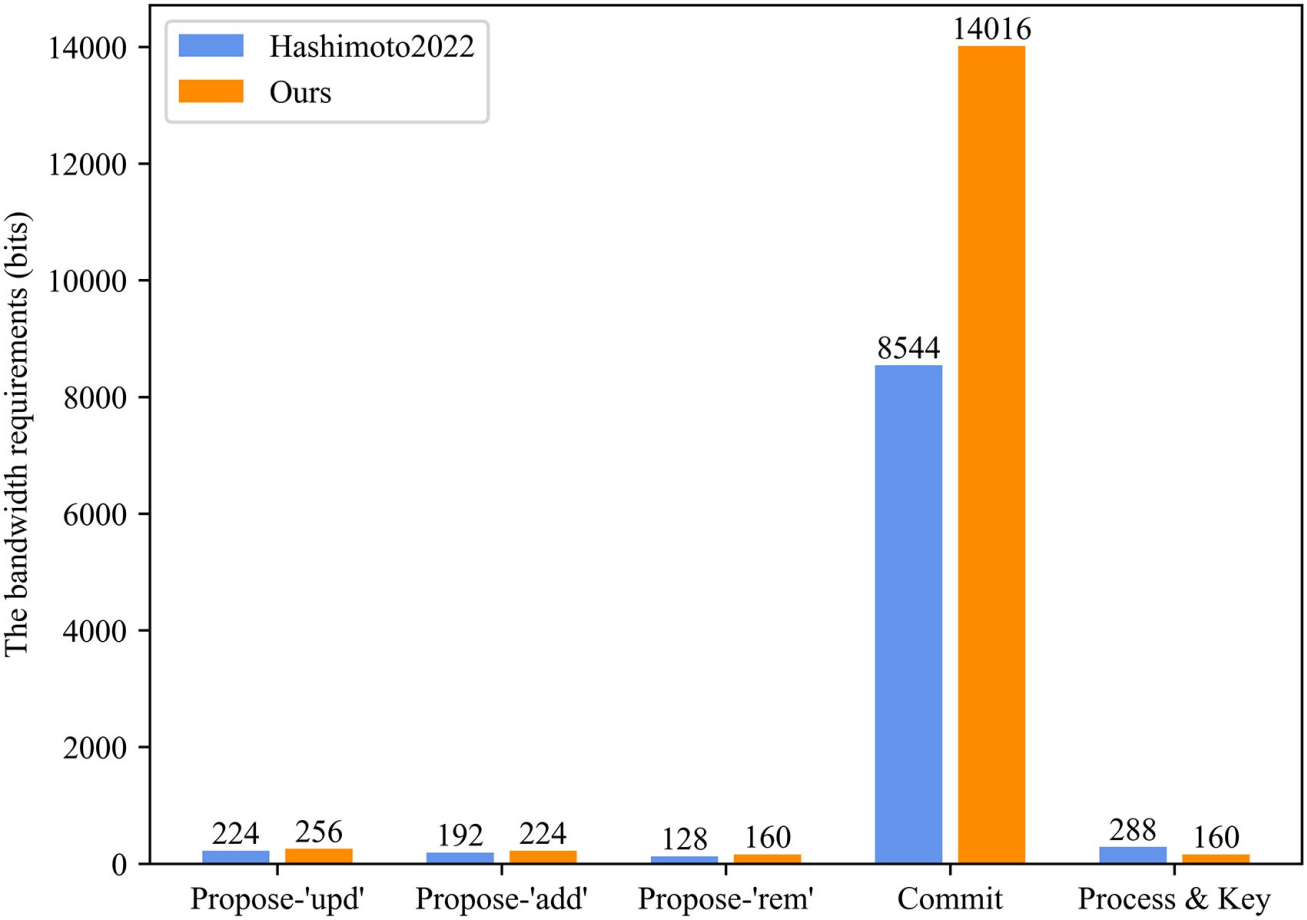
The bandwidth requirements.

From Fig 11, the ER-CGKA scheme’s bandwidth requirements are more than compared to the scheme in [8], which increased 14.29% (Propose-‘upd’), 16.67% (Propose-‘add’), 25% (Propose-‘rem’), and 60.04% (Commit stage), respectively. The reason is that we utilize pseudonym technology to achieve conditional privacy-preserving and unlinkability of the group vehicles. But at the Process & Key stage, our communication cost was reduced by 44.44% since the group vehicles don’t need to transmit signatures and commit messages. In a word, although the total communication cost in our ER-CGKA scheme increased by 55.57% than the scheme in [8], our scheme achieves more secure requirements. Thus, the result is acceptable.

### 6.3 Discussion

What’s more, to give a better presentation of this work, we compare the computation cost with schemes [41–44]. Without loss of generality, we choose Type A elliptic curve. We utilize the JPBC library to simulate cryptographic operations on a Windows 10 laptop with an AMD Ryzen 9 5950 X @4 GHz processor and 32 GB of memory. The computation time for the accomplishment of a bilinear pairing operation is 2.972 ms, and the point multiplication is 5.265 ms. The comparison results are shown in Table 5.

**Table 5:** The comparison of computation cost with schemes [41–44].

Schemes	Scheme [41]	Scheme [42]	Scheme [43]	Scheme [44]	Ours
Total computation time (ms)	2.972	2.972	5.944	32.945	5.221

As is shown in Table 5, the total computation time of our ER-CGKA scheme is less than [43, 44] and higher than [41, 42]. Specifically, schemes in [43, 44] utilize the expensive bilinear pairing operation, so their computation cost is higher. In addition, we utilized a secret sharing algorithm, an ECDSA algorithm, and many key generation algorithms to achieve PCFS and resist collusion attacks. So our computation cost is higher than [41, 42]. Overall, we achieve a better trade-off between security and performance.

## 7 Conclusion

First, we propose an ER-CGKA scheme with PCFS for IoV. Then, we prove that it is secure according to CGKA security under the ROM. It satisfies message authentication, integrity, confidentiality, non-repudiation, and unlinkability. Besides, the ER-CGKA scheme achieves conditional privacy-preserving of vehicles’ identity and PCFS of the group key. Moreover, collusion attacks by malicious vehicles can be resisted through threshold cryptography. The performance simulation results indicate that the computation cost and communication overhead are low. In the future, we will make great efforts to construct a more efficient and practical CGKA scheme for IoV and find the trade-off between communication efficiency and privacy-preserving.

## Supporting information

S1 Dataset (XLSX)

S2 Dataset (XLSX)
